# A study of surface dosimetry for breast cancer radiotherapy treatments using Gafchromic EBT2 film

**DOI:** 10.1120/jacmp.v13i3.3727

**Published:** 2012-05-10

**Authors:** Masahiro Nakano, Robin F. Hill, May Whitaker, Jung‐Ha Kim, Zdenka Kuncic

**Affiliations:** ^1^ Institute of Medical Physics, School of Physics University of Sydney Sydney NSW 2006 Australia; ^2^ Department of Radiation Oncology Royal Prince Alfred Hospital Sydney NSW 2050 Australia; ^3^ Department of Therapeutic Radiology Edogawa Hospital Edogawa‐ku Tokyo 133‐0052 Japan; ^4^ Department of Radiology The University of Tokyo Hospital Bunkyo‐ku Tokyo 113‐8655 Japan

**Keywords:** breast radiotherapy, surface doses, radiochromic film, radiation dosimetry

## Abstract

The present study quantified surface doses on several rectangular phantom setups and on curved surface phantoms for a 6 MV photon field using the Attix parallel‐plate chamber and Gafchromic EBT2 film. For the rectangular phantom setups, the surface doses on a homogenous water equivalent phantom and a water equivalent phantom with 60 mm thick lung equivalent material were measured. The measurement on the homogenous phantom setup showed consistency in surface and near‐surface doses between an open field and enhanced dynamic wedge (EDW) fields, whereas physical wedged fields showed small differences. Surface dose measurements made using the EBT2 film showed good agreement with results of the Attix chamber and results obtained in previous studies which used other dosimeters within the measurement uncertainty of 3.3%. The surface dose measurements on the phantom setup with lung equivalent material showed a small increase without bolus and up to 6.9% increase with bolus simulating the increase of chest wall thickness. Surface doses on the cylindrical CT phantom and customized Perspex chest phantom were measured using the EBT2 film with and without bolus. The results indicate the important role of the presence of bolus if the clinical target volume (CTV) is quite close to the surface. Measurements on the cylindrical phantom suggest that surface doses at the oblique positions of 60° and 90° are mainly caused by the lateral scatter from the material inside the phantom. In the case of a single tangential irradiation onto Perspex chest phantom, the distribution of the surface dose with and without bolus materials showed opposing inclination patterns, whereas the dose distribution for two opposed tangential fields gave symmetric dose distribution. This study also demonstrates the suitability of Gafchromic EBT2 film for surface dose measurements in megavoltage photon beams.

PACS number: 87.53.Bn

## I. INTRODUCTION

Radiation therapy is commonly given to breast cancer patients, often after surgery, in order to minimize the risk of local recurrence in the conserved breast, chest wall, and lymph nodes.^(^ ^1^ ^)^ The Early Breast Cancer Trialists' Collaborative Group confirmed that the combination of surgery and subsequent radiation therapy significantly reduces the risks of local recurrence and long‐term breast cancer mortality.^(^ ^2^ ^)^ However, adverse skin reactions caused by radiation therapy can be a limiting factor for breast cancer treatment. Therefore, the assessment of skin dose in the treatment planning process and the accurate measurement of skin dose are important considerations when evaluating the risk of side effects from radiation treatment.

According to the ICRP and ICRU recommendations, skin dose should be assessed at a depth of 70 μm, which corresponds to the boundary between the dermis and epidermis layers of the skin.^(^ ^3^ ^–^ ^5^ ^)^ At this depth, there is a steep dose gradient in the percentage depth dose (PDD) curve. For example, the relative dose increases from 14% to 43% within the first millimeter in a 6 MV photon beam with a field size of $10\;\times \;10\;{\text{cm}}^{2}$, making the measurement and estimation of surface dose difficult.^(^ ^6^ ^)^

While modern radiotherapy treatment planning systems (TPS) are, in most cases, able to accurately predict doses to a patient, a number of studies have demonstrated that surface and near‐surface doses estimated by TPSs are inaccurate. This is recognized by the AAPM in their document on commissioning TPSs, which suggests an acceptability criteria of up to 20% in the build‐up region of X‐ray depth dose curves between measurements and TPS calculations.^(^ ^7^ ^)^ Devic et al.^(^ ^3^ ^)^ indicated that most TPS apply the beam data measured at the depth of dose maximum and beyond to calculate all parts of the dose distribution, including the build‐up region, and the surface dose is estimated by extrapolating measured data toward the surface with fitting functions.

For curved structures such as the breast, chest, as well as head and neck, skin dose assessment becomes even more complicated. Court et al.^(^ ^8^ ^)^ reported inconsistencies of up to 27% between skin dose assessment estimated by the Eclipse TPS (Varian Medical Systems, Palo Alto, CA) and dose measured by MOSFET dosimeters for 6 MV and 10 MV photon beam fields in the case of a semi‐cylindrical water equivalent phantom. Chung et al.^(^ ^9^ ^)^ reported overestimation by up to 18% of the surface dose on a semi‐cylindrical phantom representing a head and neck cancer patient by the Pinnacle TPS (Philips Medical Systems, Andover, MA), compared to measurements using Gafchromic HS film.

Surface doses are increasingly being measured with radiochromic film, which has several advantageous features for dosimetry, such as its high planar spatial resolution, low spectral sensitivity, tissue equivalency, self‐development, and concise usage.^(^ ^10^ ^,^ ^11^ ^)^ A comprehensive study on surface doses measured using radiochromic films was done by Devic et al.,^(^ ^3^ ^)^ who reported PDD measurements within the first millimeter of a water phantom for a 6 MV photon beam using a variety of radiochromic films and a few other dosimeters. That study also determined correction factors to scale the dose measured at the effective point of measurement for a given detector to the ICRP skin dose at a depth of 70 μm. Several studies have investigated the energy response of different film dosimeters over a wide range of photon energies, and Gafchromic EBT film (International Specialty Products (ISP), Wayne, NJ) has been demonstrated to be a film dosimeter with minimal photon energy dependence.^(^ ^12^ ^,^ ^13^ ^)^ In 2009, the manufacturer ISP released EBT2 film as a new version of Gafchromic film, which has a different configuration of its multiple layers from that of its predecessor.^(^ ^14^ ^,^ ^15^ ^)^ Kairn et al.^(^ ^16^ ^)^ confirmed that EBT2 film keeps up the utility of previous EBT film with good water‐equivalence and little angular dependence.

In this study, surface doses on rectangular phantom setups and on phantoms with curved surfaces have been investigated quantitatively. In the case of rectangular phantoms, surface doses of homogenous and inhomogeneous phantom setups with water equivalent phantom and lung equivalent material were measured. In the case of phantoms with curved surfaces, surface doses of a cylindrical CT phantom and chest‐simulated phantom with bolus materials were investigated. The present study also further validates the use of Gafchromic EBT2 film to measure surface doses in 6 MV photon beams.

## II. MATERIALS AND METHODS

### A. Linear accelerator and field arrangements

In this study, all measurements were performed using a 6 MV photon beam produced by a Varian Clinac 21EX linear accelerator (Varian Medical Systems, Palo Alto, CA). The field size of the photon beam was $10\;\times \;10\;{\text{cm}}^{2}$, unless otherwise stated. The irradiation fields included: an open field; fields with physical or hard wedges (HW) of 15°, 30°, 45°, and 60°; fields with enhanced dynamic wedges (EDW) of 15°, 30°, 45°, and 60°; and fields with several programmed multileaf collimator (MLC) movements. The MLC fields were dynamic (DMLC) square fields with a fixed gap varying from 4 mm to 4 cm to swipe across the field. For the fields with physical wedges, the radiation output is decreased due to the beam attenuation caused by the physical wedge; hence, specific wedge factors for each wedge angle were applied in the treatment planning calculations in order to deliver the same dose for each irradiation.^(^ ^10^ ^)^

### B. The Attix parallel‐plate ionization chamber

The Attix parallel‐plate ionization chamber, RMI model 449 (RMI, Middleton, WI) has been recognized as the gold standard of surface and near surface dosimetry for megavoltage photon beams.^(^ ^3^ ^,^ ^17^ ^–^ ^19^ ^)^ It enables highly accurate measurements of surface dose due to its very shallow effective point of measurement of 48 μm.^(^ ^3^ ^)^ In the present study, the Attix chamber was used to measure surface and near surface doses under the homogenous water‐equivalent rectangular phantom setups. These measurements were compared with corresponding measurements made with Gafchromic EBT2 film. The Attix chamber was connected to a PTW UNIDOS (PTW, Freiburg, Germany) electrometer.

### C. Gafchromic EBT2 film and its calibration procedure

Radiochromic film has been shown to be suitable for dose measurements in surface and near surface regions due to its energy independence, tissue equivalency, and high sensitivity.^(^ ^3^ ^,^ ^15^ ^,^ ^20^ ^)^ The present study employed radiochromic Gafchromic EBT2 film (hereafter called EBT2 film) as a principal dosimeter to measure surface doses.^(^ ^21^ ^)^ The lot number of the film used was F04090901, and a sheet of film (of area $20.3\;\times \;27.9\;{\text{cm}}^{2}$) was cut into $2\;\times \;2\;{\text{cm}}^{2}$ pieces and marked in order to distinguish the side of the polyester over‐laminate layer. This side was positioned face up for all measurements to ensure the active layer was located closer the surface. The 30 μm thick active layer is positioned 80 μm from the surface of the film in this orientation, as opposed to 175 μm in the opposite side. In this orientation, the active layer was located at the depth quite close to the ICRU skin dose of 70 μm.

Surface doses for 6 MV X‐rays were measured using the EBT2 film. These results were then normalized by the maximum dose measured in a water equivalent phantom under reference conditions (15 mm depth, source to surface distance (SSD) of 100 cm and a field size of $10\;\times \;10\;{\text{cm}}^{2}$) to obtain relative surface doses.

An X‐Rite densitometer, model 361T (X‐Rite Inc., Grandville, MI), with 2 mm aperture was used to read the optical densities (OD) of the irradiated films. In order to obtain precise ODs, the irradiated films were left to self‐develop for 24 hours or greater, and the densitometer was switched on 15 minutes prior to readout to allow for lamp warm‐up.^(^ ^21^ ^,^ ^22^ ^)^ All EBT2 film pieces were read prior to irradiation in order to obtain the background OD of each piece. In order to minimize the effect of sensitivity variation with position within the film, postirradiation ODs were read from three different points on each piece of film. We then define the netOD as irradiated, or exposed OD (${\text{OD}}_{\text{exp}}$) subtracted by background, or unexposed OD (${\text{OD}}_{\text{unexp}}$), according to Devic et al.^(^ ^23^ ^,^ ^24^ ^)^

(1) $$netOD^{i}(D_{j})=OD_{\text{exp}}^{i}(D_{j})-OD_{\text{unexp}}^{i}(D_{j})$$

The calibration datasets which enable transformation from netODs to doses in Gy were obtained for each set of film pieces from one film sheet, and for each measurement. A third order polynomial curve fit was applied to each calibration dataset, based on measurements at reference conditions for a 6 MV X‐ray beam. Calibration data points covered the entire range of measurement points, to avoid the need for extrapolation.

### D. Uncertainty analysis for Gafchromic EBT2 film dosimetry

The uncertainties in EBT2 film measurements were derived in accordance with the ISO methodology as described in the IAEA TRS‐398 dosimetry protocol.^(^ ^20^ ^,^ ^25^ ^–^ ^28^ ^)^ Measurement uncertainties are estimated as relative standard uncertainties, and the sources of standard uncertainties are sorted into Type A and Type B. The factors contributing to uncertainties in our surface dose measurements using EBT2 film are listed in Table 1.

**Table 1:** Standard uncertainty in the measurement of surface or near surface dose using Gafchromic EBT2 film.

*Source of Uncertainty*	*Uncertainty Type*	*Standard Uncertainty (%)*
1. OD measurement over pixels	A	0.7
2. OD measurement over multiple film pieces	A	1.8
3. Linac X‐ray output reproducibility	B	0.2
4. EBT2 film best‐fit calibration curve	B	1.7
5. Setup repeatability of the phantom and film pieces	B	0.1
6. Dose output accuracy	B	0.6
7. Film homogeneity	B	1.9
Total Uncertainty		3.3%

### E. Surface dose measurement on rectangular phantom setups

**Figure 1:**
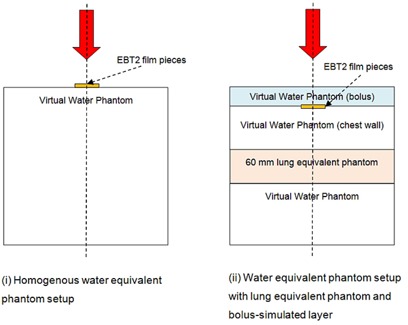
Rectangular phantom setups: (i) homogeneous phantom; (ii) heterogeneous phantom with lung equivalent material beneath additional layers of varying thickness representing bolus and chest wall.

In order to understand the basic characteristics of surface doses, the relative dose measurements of surface doses were conducted with two rectangular phantom setups with and without heterogeneities. The phantom setups consisted of a combination of Virtual Water (Standard Imaging Inc., Middleton, WI) having a physical density of $1.03\;g/{\text{cm}}^{3}$ as a water equivalent phantom, and Gammex 455 lung equivalent material (Gammex Inc., Middleton, WI) having a physical density of $0.300\;g/{\text{cm}}^{3}$ as a lung equivalent phantom. The two phantom setups were: (i) homogenous Virtual Water phantom setup, and (ii) Virtual Water phantom setup with lung equivalent phantom and bolus. The SSD for all phantom setups was 100 cm. Figure 1 shows a schematic diagram of the two rectangular phantom setups.

In phantom setup (i), surface doses were measured using both the Attix parallel‐plate chamber and EBT2 film and results were compared with each other. The Attix chamber measurements were adjusted using the correction factor suggested by Devic et al.,^(^ ^3^ ^)^ to obtain the dose at the ICRP skin depth of 70 μm.

In phantom setup (ii), 60 mm of lung equivalent material was inserted in the rectangular Virtual Water phantom setup. The Virtual Water phantom above the lung layer simulated layers of bolus material and chest wall of varying thicknesses. The thickness of chest wall‐simulated Virtual Water phantom varied from 2 mm to 50 mm, while the thickness of the bolus‐simulated Virtual Water phantom was 0, 5, 10, and 15 mm. The EBT2 film pieces were placed between the bolus‐simulated and chest wall‐simulated layers of Virtual Water phantom. The surface doses thus obtained were normalized by the maximum dose at 15 mm depth under the reference conditions specified in Section C above.

### F. Surface dose measurement on a cylindrical CT phantom

**Figure 2:**
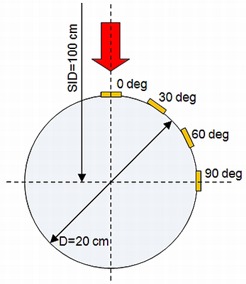
Geometrical overview of the cylindrical CT phantom setup and positions of EBT2 film pieces.

Surface doses on a cylindrical CT phantom were measured using EBT2 film pieces in order to investigate the dose distribution on the curved surface with and without bolus. The cylindrical CT phantom used was Catphan (The Phantom Laboratory, Salem, NY), which had a 20 cm diameter and was placed in an isocentric geometry with a source‐to‐isocenter distance (SID) of 100 cm. EBT2 Film pieces were placed at four different angles on the curved surface, from 0° to 90° in 30° increments, as shown in Fig. 2. Surface doses with bolus thicknesses of 5, 10, and 15 mm were measured. Corresponding surface doses without bolus were also measured. The field size in this case was $20\;\times \;20\;{\text{cm}}^{2}$ from a gantry angle of 0° in order to include all film pieces in the irradiation field. The depth of maximum dose was corrected by the output factor for the field size of $20\;\times \;20\;{\text{cm}}^{2}$ and SSD factor, and surface doses were normalized by the corrected maximum dose to obtain the relative surface dose under our reference conditions.

### G. Surface dose measurement on chest‐simulated phantom

**Figure 3:**
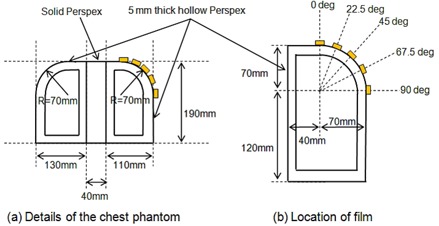
Geometrical details of the Perspex chest phantom and the location of Gafchromic EBT2 film pieces.

**Figure 4:**
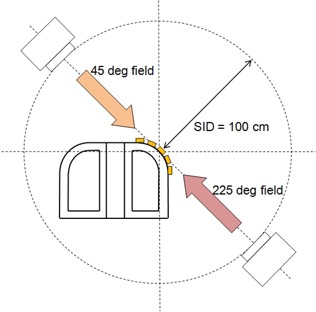
Schematic diagram of surface dose measurement on the Perspex chest phantom.

To investigate doses on the curved surface of a breast cancer patient in a tangential field setup, surface doses on a chest‐simulated Perspex phantom with and without 6 mm thick bolus were measured using EBT2 film pieces. The details of the phantom and the schematic diagram of the irradiation geometry are shown in Figs. 3 and 4, respectively. The phantom consisted of three parts: the mediastinum, and two lung parts. The mediastinum was represented by 40 mm thick solid Perspex. The two lung parts were made by 5 mm thick Perspex with a hollow cavity inside. The width of the left lung part was 110 mm, while that of the right was 130 mm to represent the asymmetry of those structures. EBT2 film pieces were placed at five different positions on the curved surface of the left lung, at 0°, 22.5°, 45°, 67.5°, and 90°. The irradiation angles were directed at 45° for a single tangential field, and at 45° and 225° for two opposing tangential fields, as shown in Fig. 4. The field size was $10\;\times \;10\;{\text{cm}}^{2}$.

## III. RESULTS

### A. Dose measurement in the build‐up region using the Attix parallel‐plate chamber

**Figure 5:**
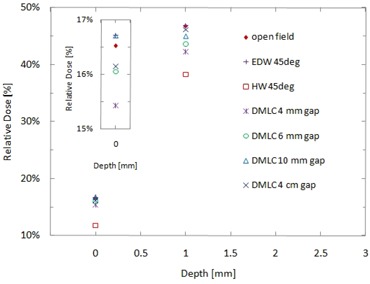
Relative doses measured with an Attix chamber and with EBT2 film in the build‐up region of a homogeneous water phantom for wedge fields inclined at 45° (enhanced dynamic wedge (EDW) and hard wedge (HW)) and for dynamic MLC (DMLC) fields with varying MLC gap thicknesses. The inset shows a zoom‐in of the dose measurements near 0 mm.

Figure 5 presents our results for doses measured with the Attix chamber in the build‐up region of the homogeneous water equivalent rectangular phantom for the open field, 45° EDW and physical wedge, and for the dynamic MLC fields. The surface dose and the dose at 1 mm depth for the open field were 16.5% and 46.8%, respectively, in good agreement with the corresponding results reported by Devic et al.^(^ ^3^ ^)^ The doses of the 45° physical wedge field show the lowest values both at the surface and at 1 mm depth. Regarding MLC fields, as the width of MLC aperture increased from 4 mm to 4 cm, the dose increased slightly both at the surface and at 1 mm depth.

**Figure 6:**
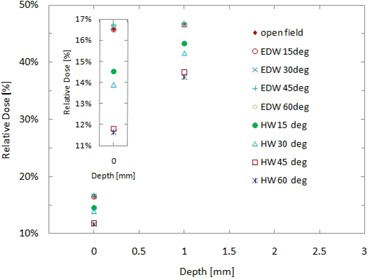
Relative doses in the build‐up region with various wedge techniques. The inset shows a zoom‐in of the surface dose measurements.

Figure 6 shows the relative doses of several EDW and physical wedge fields in the build‐up region. The difference between open field doses and EDW doses were within 0.3%. The doses for the four different physical wedge fields were less than the open field doses, and the surface dose decreased with increasing wedge angle (i.e., increasing wedge thickness).

### B. Uncertainty analysis of Gafchromic EBT2 film surface dosimetry

The total uncertainty in the surface dosimetry using EBT2 film in this study was estimated to be approximately 3.3%, as shown in Table 1. This uncertainty was calculated using the ISO methodology.^(^ ^25^ ^,^ ^26^ ^,^ ^28^ ^,^ ^29^ ^)^ The main factors contributing to this value of uncertainty are: the OD measurements over multiple film pieces, the EBT2 film dose calibration curve, and the film inhomogeneity. Our uncertainty estimate for EBT2 is slightly less than the 4%–6% estimated by Richley et al.,^(^ ^22^ ^)^ Lindsay et al.,^(^ ^14^ ^)^ and Hartmann et al.^(^ ^21^ ^)^ There are three contributing factors to our lower measurement uncertainty estimate: the stabilization of the film manufacturing process in the more recent batches of EBT2 film, as discussed by Lindsay et al.; our use of a densitometer instead of a film scanner for OD readout; and our careful positioning of the EBT2 film with its active layer facing towards the X‐ray source, which is a more accurate method, particularly for determining surface dose.

### C. Surface dose measurement on a homogenous phantom

**Figure 7:**
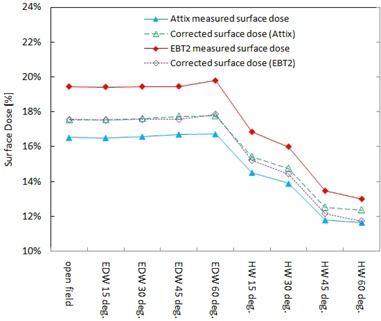
Surface doses on a homogeneous phantom measured by Attix chamber and EBT2 film, and corresponding skin doses corrected to a depth of 70 μm, for various field configurations.

Figure 7 shows the measured surface doses on the rectangular homogenous Virtual Water phantom setup using EBT2 film pieces, as well as an Attix parallel‐plate chamber. The measured surface doses using the Attix chamber and EBT2 film for the open field are 16.5% and 19.4%, respectively. Corrected skin doses measured by the Attix parallel‐plate chamber and EBT2 film are also shown in Fig. 7 as dot‐dashed lines. The corrected doses for the Attix chamber results were obtained by applying the correction factor provided by Devic et al.,^(^ ^3^ ^)^ which is 1.062, to obtain ICRP skin dose at the depth of 70 μm. The correction factor of 0.903 for EBT2 film was derived experimentally from the corrected skin dose value of the open field measured by the Attix chamber divided by the measured surface dose value by EBT2 film.

### D. Surface dose measurement in a phantom containing lung equivalent material

**Figure 8:**
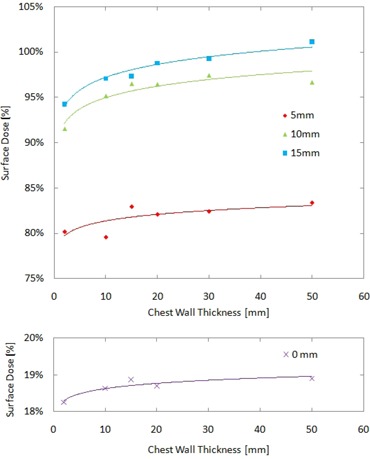
Surface doses on a heterogeneous phantom containing a 60 mm lung layer for different bolus thicknesses (0, 5, 10, and 15 mm) and varying chest wall thickness (from 2 mm to 50 mm).

Figure 8 shows the surface doses for the phantom containing 6.0 cm of lung material with and without bolus at the surface. The solid curves in the plot present a guide for the eye. The surface doses without bolus show a slight increase as the thickness of Virtual Water above the lung equivalent material was increased, from 18.3% for 2 mm Virtual Water thickness up to 18.9% for 50 mm thickness. These surface doses are slightly lower than the surface dose of 19.4% for the homogenous phantom setup and this difference can be attributed to a slight reduction in backscatter leading to a reduction of surface dose.

The surface dose results with three different bolus layer thicknesses are also shown in Fig 8. The results show an increase in surface dose with increasing chest wall thickness, although the total dose is higher due to the presence of the bolus material at the surface. The maximum surface dose of 101.1% is found at the chest wall thickness of 50 mm with 15 mm bolus. This can be attributed to the Virtual Water thickness of 50 mm for which the EBT2 films were almost in a full scattering condition.

### E. Surface dose measurement on a cylindrical CT phantom

**Figure 9:**
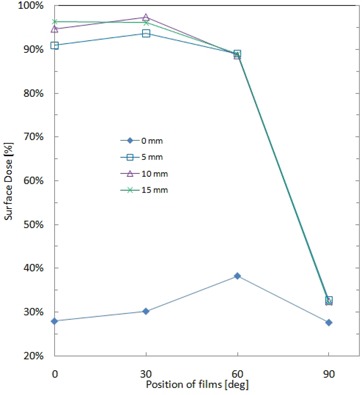
EBT2 measured surface doses on the cylindrical CT phantom with different bolus thicknesses (0, 5, 10, and 15 mm).

Measured surface doses on the cylindrical CT phantom of 20 cm diameter in an open field of $20\;\times \;20\;{\text{cm}}^{2}$ are shown in Fig. 9. In the case without bolus, the surface dose increases from 27.9% to 38.2% as the angle of the measurement position increases from 0° to 60°. In the case of surface doses with bolus materials of various thicknesses, doses at 0° and 30° for all bolus thicknesses were 94.9% ± 3.9%, and showed 3.4 times increase compared to the case without bolus.

### F. Surface dose measurement on a chest phantom

**Figure 10:**
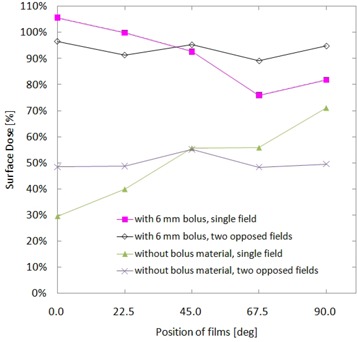
Surface doses on Perspex chest phantom for single and two opposed tangential field setups, with and without 6 mm bolus.

Figure 10 shows the results of surface dose distribution on the chest‐simulated Perspex phantom. Surface doses with and without 6 mm thick bolus, and the results of single tangential field and two opposed tangential fields are shown. In the case of the single tangential irradiation, distributions of the surface dose with and without bolus show an opposite pattern. Without bolus, the dose increases from 29.5% up to 71.0% as the angle of measurement position increases from 0° to 90°, and the peak of the dose was observed at 90° which locates the opposite side of radiation source. In contrast, the peak dose of 105.6% was observed at 0° in the case with bolus and the surface dose decreases to 75.9% at 67.5°. This is attributed to the presence/absence of bolus material to produce scattered electrons.

The surface dose of two opposed tangential fields distributed symmetrically around the dose at 45° in cases both with and without bolus were investigated. The surface doses ranged from 48.3% (at 67.5°) to 55.2% (at 45°), and from 89.1% (at 67.5°) to 96.6% (at 0°) for without bolus and with bolus, respectively. The surface doses with bolus show 1.7 times increase at 45° and 1.9 times increase at 0° and 90° compared to the case without bolus, which is to be expected due to the extra buildup.

## IV. DISCUSSION

### A. Surface dose measurement on homogenous phantom setup

In a measurement in homogenous rectangular phantom setups, surface doses for the open field measured by the Attix parallel‐plate chamber and EBT2 film show good agreement with the study of Devic et al.,^(^ ^3^ ^)^ as shown in Fig. 7. This confirms that EBT2 film is suitable for accurate surface dose measurements in 6 MV photon beam fields. The difference in the surface dose between the open field and physical wedge fields shown in Fig. 6 can be attributed to two factors: reduction of secondary electrons produced at the linac head due to the presence of the wedges, and the change in beam spectrum of photons and electrons resulting from interactions with high atomic number materials in the physical wedges.

The corrected doses for the four physical wedge fields show a maximum difference between the two dosimeters of up to 0.6%. These results are in good agreement and the differences are small compared to the calculated uncertainty in EBT2 film measurements.

### B. Surface dose measurement on a phantom containing lung equivalent material

Regarding surface dose on a phantom containing lung material, different inclination was observed between measurements without bolus and with bolus, as shown in Fig. 8. The results without bolus indicate that varying chest wall thickness causes little difference of 0.6% in surface dose, and inhomogeneity between Virtual Water and lung material phantom does not appreciably affect surface dose. In contrast, surface doses with bolus showed an increase with increasing chest wall thickness. The variation of surface dose with varying chest wall thickness was 3.7%, 5.9%, and 6.9% for 5, 10, and 15 mm bolus thickness, respectively. This indicates that the thickness of chest wall leads to the difference in surface dose in situations where bolus is present. In this case, it is important to know the actual depth of a tumor in order to choose an adequate bolus thickness. If the tumor depth is shallow, bolus material placed directly on a patient is quite effective.

### C. Surface dose measurement on a cylindrical CT phantom

Surface dose measured on the cylindrical CT phantom, shown in Fig. 9, suggests dominance of lateral scattered radiation in dose distribution on curved surface. In the case without bolus, lower doses are observed at 0° and 30°, which are 27.9% and 30.2%, respectively, due to the absence of build‐up material. The peak of the surface dose is observed at 60°, which is due to the presence of phantom material above this point that produces lateral scattered radiation which increases the surface dose. The surface dose at 90° was 27.6% despite the film being positioned in the penumbral region. This may be due to the lateral scattered radiation observed at 60°, which also dominates the surface dose at 90°. In the case with bolus materials, doses at 60° and 90° showed only small changes in surface dose due to the difference of bolus thickness. This indicates that surface doses at 60° and 90° are mainly from lateral scatter within the phantom.

In both cases with and without bolus, the measured surface doses from 0° to 60° show a similar trend to the measurement results of Quach et al.^(^ ^30^ ^)^ using Gafchromic MD‐55‐2 film. In particular the entrance dose (at 0°) without bolus shows very good agreement with the result of Quach et al., which was 28%. The only difference was observed in the case of the results at 90° which are attributed to the difference of the radius of the curved surface. The measurement position at 90° in the present study was located almost in the penumbra, whereas the position at the same angle in the measurement of Quach et al. seemed to be located totally inside of the umbra.

### D. Surface dose measurement on a chest phantom

The result of surface dose distribution on the chest‐simulated phantom, shown in Fig. 10, emphasizes the difference of the presence/absence of bolus material. Each of measured results demonstrates unique distribution of surface dose for each irradiation field. Hsu et al.^(^ ^18^ ^)^ evaluated surface doses in a similar geometry using an anthropomorphic phantom and observed similar symmetrical distribution of surface doses. Their results ranged from 45% to 62% and from 81% to 106% for without bolus and with 2 mm bolus, respectively. The differences between the study by Hsu et al. and the current study can be primarily attributed to the structure of the phantom in this study which consisted of 5 mm thickness shell of Perspex and a hollow cavity inside. However, it was confirmed that a more uniform distribution of surface doses were provided by two opposed fields.

## V. CONCLUSIONS

In this work, surface doses have been determined for a wide range of geometries and fields. The surface and near‐surface dose measurements on a homogenous, rectangular slab Virtual Water phantom using the Attix parallel‐plate chamber showed a steep dose increase in the buildup region. Doses for the open field and enhanced dynamic wedge fields were consistent with each other, while doses for the physical wedge fields were slightly lower. The doses measured with Gafchromic EBT2 film were in good agreement with the Attix parallel‐plate chamber results. From this, the Gafchromic EBT2 film is recommended as being suitable and accurate for surface dosimetry.

Our surface dose measurements on a rectangular water equivalent phantom with 60 mm lung material showed an increase in surface dose with increasing chest wall thickness. Measurements with three different bolus thicknesses presented up to 6.9% increase as thickness of chest wall increased, while an increase in surface dose without bolus was just 0.6%.

The surface dose measurements on a cylindrical phantom and on the curved surface of a chest phantom with several bolus thicknesses indicate the important role of the presence of bolus material if the clinical target volume (CTV) is close to the surface. The surface dose measurement on the cylindrical phantom suggested that the surface doses at 60° and 90° are mainly caused by the lateral scatter from the material inside the phantom. In the case of single tangential irradiation onto the curved surface chest phantom, the distribution of the surface dose with and without bolus material showed the opposite pattern. In contrast, the dose distribution for the two opposed tangential fields gave a totally symmetric dose distribution.

Throughout this study, Gafchromic EBT2 film was found to be suitable for surface dose measurements. We suggest that it can be used for *in vivo* surface dosimetry in megavoltage photon beam fields.

## ACKNOWLEDGMENTS

The authors would like to acknowledge the National Breast Cancer Foundation (Australia) for financial support of this study. They would also like to thank the Department of Radiation Oncology, Royal Prince Alfred Hospital, Sydney (Australia) for providing access to equipment.
